# Application Research: Big Data in Food Industry

**DOI:** 10.3390/foods10092203

**Published:** 2021-09-17

**Authors:** Qi Tao, Hongwei Ding, Huixia Wang, Xiaohui Cui

**Affiliations:** 1 Key Laboratory of Aerospace Information Security and Trusted Computing, Ministry of Education, School of Cyber Science and Engineering, Wuhan University, Wuhan 430072, China; qtao17@whu.edu.cn (Q.T.); hwding@whu.edu.cn (H.D.); 2 Hubei Provincial Institute for Food Supervision and Test, Wuhan 430223, China; whx097@126.com

**Keywords:** food industry, big data, application prospect, sustainable development

## Abstract

A huge amount of data is being produced in the food industry, but the application of big data—regulatory, food enterprise, and food-related media data—is still in its infancy. Each data source has the potential to develop the food industry, and big data has broad application prospects in areas like social co-governance, exploit of consumption markets, quantitative production, new dishes, take-out services, precise nutrition and health management. However, there are urgent problems in technology, health and sustainable development that need to be solved to enable the application of big data to the food industry.

## 1. Introduction

Consumers are no longer satisfied with having enough to eat; food quality has become a key factor in determining consumer choice [1], and their demands and preferences change with the season, time, weather, mood, and other factors [2]. However, food choice is a luxury not every person enjoys. The Food and Agriculture Organization (FAO) of the United Nations reported that 88% of countries face a serious malnutrition burden and so has issued healthy dietary guidelines that cover a wide range of food and nutrition (http://www.fao.org/nutrition/education/food-dietary-guidelines/regions/countries/united-states-of-america/en/ (accessed on 2 September 2021)). Traditional food science has been unable to satisfy increasing demand for food in a world where “healthy nutrition” has overtaken “well-fed” as the predominant paradigm of consumption (https://baijiahao.baidu.com/s?id=1681293145359468742&wfr=spider&for=pc (accessed on 2 September 2021)) [3]. However, big data offers food science a new means of scientific analysis [4].

The food supply chain is composed of economic stakeholders from primary producers to consumers. It has the characteristics of large volume, many links, wide distribution, diverse types, and scattered data, and it is becoming more complex. Millions of tons of food move around the world every year, so no enterprise can promise that every risk node on the production line is absolutely safe. Any flaw in the supply chain could bring a disaster and huge regulatory difficulties for government departments. However, big data provides a solution to regulatory difficulties [5] by helping enterprises understand consumer demand better and uncover food industry trends through big data analysis. The food industry collects large datasets through real-time monitoring and can improve food safety if analyzed in conjunction with sample data [6]. When industry data is combined with data on consumer dietary behavior, food enterprises can optimize their investment and adjust the direction of research and development in a timely manner. [7].

**Figure 1:**
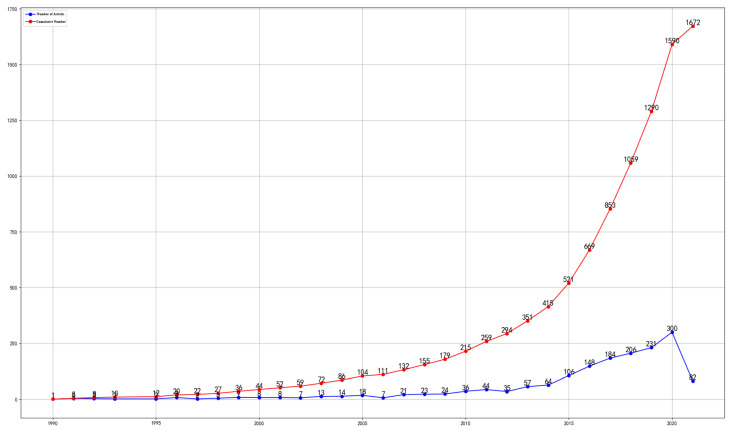
Research progress of data in the food field. From 1990 to 2010, the research papers of big data on food grew at a rate of 100% every five years, and since 2010 it has grown by nearly 300% every five years.

**Figure 2:**
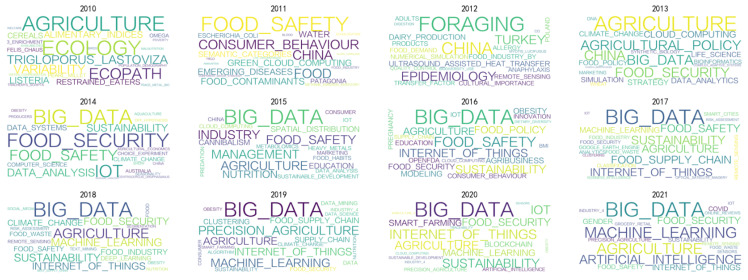
Analysis of the research direction of the data from 2010 to 2021 in the food field. Since 2013, food security and big data have become a focus of researchers interested in the potential value of big data on food. The research focuses on IoT-based data collection and its application to smart farming, supply chain management, food nutrition, and sustainable development.

This paper uses bibliometrics to analyze the research progress of big data in the food field. According to Bradford’s Law, a small number of core journals collect enough information to reflect the latest and most important advances in science and technology. The database of Web of Science Core Collection contains more than 12,000 core journals from more than 250 subject areas. It defined the search topic “Food & Big Data” and selected 1672 papers from its database. The research progress of big data on food is shown in Figure 1 It has increased significantly since 2014 because USD 35.8 billion was invested in global agrifood from 2010 to 2019, and after 2014 the scale of financing grown rapidly (https://agfunder.com/research/agfunder-agrifood-tech-investing-report-2019/ (accessed on 2 September 2021)). This increased capital investment promoted research into big food data, and the rapidly rising trend is from 2010 to 2021 is shown in Figure 2. China’s food industry has attracted global attention since 2012 because the country’s new government leaders stressed that they would pay more attention to food safety (http://www.xinhuanet.com/politics/2017-01/03/c_1120239001.htm (accessed on 2 September 2021)). As the second largest economy and the world’s largest trading country, China has great international influence. (https://www.brookings.edu/research/chinas-influence-on-the-global-middle-class/ (accessed on 2 September 2021)). Big data has been one of the focuses of research since 2013, mainly in food safety, food security and agriculture. Its application to food safety may still be in its infancy, but it is affecting the entire supply chain. The literature contains analyses on the feasibility and need for big data in the food industry [4,6], but there are no in-depth analyses. Therefore, this paper will mainly discuss the following three aspects in depth.

- The major sources of big data in food industry and its challenges.
- The market application trends of big data in the food industry.
- The main challenges to applying big data.

The authors of this paper hope to help researchers develop a deeper understanding of the research progress of big data in the food field and to provide guidance for further research.

## 2. Big Data in Food Industry

Big data sources of food mainly include regulatory, food enterprise (including data generated at every link of the industrial chain from planting to restaurants), and media data (including food-related news, video, pictures and audio). High-quality big data analysis can help develop the food industry, whereas analyses from low-quality data can adversely affect managers’ prediction of market demand [8], and social stability [9].

### 2.1. Food Regulatory Data

Food regulatory data usually includes department regulatory and product sampling data. Marvin [4] has detailed public information about food safety supervision and sampling inspection in various countries. This information includes, reports on animal and plant disease monitoring, hazards, food-borne diseases, which provided support for researchers of deep-risk information. Rapid Alert System of Food and Feed (RASFF) is a commonly used online food safety database for industry and scientific research in the European Union (EU). Food safety databases in other countries include the Import Rejection Report (IRR) and the Inspection Classification Database (ICD) in the U.S. and the State Administration for Market Regulation (SAMR) alerts in China [5]. With the increasingly close connection between countries, the trend of “table globalization” has become increasingly prominent. In 2017, the amount of food China imported from Australia, the United States, Japan, Germany, Southeast Asia, and other countries exceeded RMB 1.5 trillion (https://www.askci.com/news/chanye/20171212/084457113784.shtml (accessed on 2 May 2021)). As shown in Table 1, the SAMR usually shares its sampling inspection results of imported and exported food on government websites, which allows consumers to know the quality of food on the market. The U.S. government shares food sample analysis reports through the FSIS system. The EFSA database contains data on food consumption habits and patterns across the European Union. Such statistical data allows users to quickly screen long-term and acute exposures to potentially hazardous substances in the food chain. In addition, the World Health Organization (WHO) established the Global Environmental Monitoring System (GEMS/Food) in 1976, in which participating institutions submit data on food pollutant concentrations and set up data centers to help governments, the Codex Alimentarius Commission(CAC) and other institutions to assess trends in food contaminants [10]. In 2015, the WHO integrated data from the fields of agriculture, food, public health and economics to build a big data services platform for food safety (https://www.who.int/foodsafety/foscollab/en/ (accessed on 2 May 2021)) to improve risk monitoring.

**Challenges.** The sharing and circulation of data among food regulatory departments is conducive to the construction of intelligent supervision of the food supply chain [11]. However, there are several challenges.

- **Limited shared data.** Government departments have not been able to fully disclose detailed monitoring and sampling data, leading to the repeated testing of product quality indicators by enterprises, which has resulted in serious waste of social resources and increased operating costs. How to encourage various departments to share data is an important research direction.
- **Lack of system standards.** Due to a lack of system standards, the independence of departments leads to the relative independence of food supervision system. In addition, the limitation of responsibilities further intensifies the independence of departments. So, it is urgent that a new mode of interdepartmental data sharing be explored.
- Due to inconsist food standards, **there are differences in the names and categories of the same food**, which is an obstacle to data sharing. The Estonian government has proposed X-Road architecture of data sharing among the basic sectors [12], and a few European governments will also be involved in international data sharing [13].

### 2.2. Food Enterprise Data

The food industry chain is composed of enterprises from agriculture, fishing, processing and restaurant and is characterized by many links and wide distribution. At present, all agricultural machinery is electronically controlled to improve operational performance [14,15]. Cloud computing, the Internet of Things, big data and blockchain can integrate isolated production lines in the food supply chain into data-driven interconnected intelligent systems. Through semantic active technology, each operation is automatically integrated, improving the efficiency of precision agriculture and enterprise management [16]. Using sensors and drones to collect data on weather, geography, and animal and crop behavior can help farmers optimize crop planting and animal growth cycles. Intelligent devices capture actionable data and make decisions that reduce equipment downtime [17].

In recent years, research on the IoT in the food industry has promoted the diversification of the IoT platform to address market needs, [18,19] different monitoring models [20], and unbalanced energy consumption [21]. IoT-integrated applications will help food companies create new data sources. Industry 4.0 not only promotes the rapid development of Agriculture 4.0, but also enables enterprises to transmit real-time information to identify and meet the changing demands of stakeholders [15]. According to the Eurostat report (https://www.brookings.edu/research/chinas-influence-on-the-global-middle-class/ (accessed on 2 September 2021)), the application of smart agriculture will save 4–6% of agricultural costs and increase market value by 3% by 2026. The application of big data can not only enable businesses to deal with challenges in food production, but also to obtain more affordable raw materials to reduce production costs [14]. It also promotes the development of smart agriculture, which helps save water [22], preserve soil, limit carbon emissions [23] and improveproductivity [24]. Smart agriculture provides an opportunity for farmers, service providers, government and other stakeholders (such as financial institutions, investors, traders) to share their experiences in optimizing the agricultural supply chain with the production sustainability [25].

**Challenges.** The food industry can benefit from big data services, but there are challenges that need to be addressed, including data fairness such as the searchability, accessibility, interoperability, and reusability of shared data, and a lack of information standards and data processing technology.

- **Lack of Information standards.** (a) The lack of standardized protocols has created incompatibilities among information management systems [26]; (b) The development of the IoT is still in its initial stages. As IoT manufacturers develop independently, the data generated may be difficult to interpret and share. In addition, there are other problems such as IoT security in food safety. Any insecure IoT node in the food supply chain can be a weak link in the entire system.
- **Immature processing of food big data.** Although cloud computing has been used by many organizations, its appplication to big data regarding food safety is still in its infancy. There as also problems such as system scalability, data fairness, data security, and legal issues, which have not been adequately addressed. Blockchain technology is expected to bring a safer and more transparent food supply chain, but it is still immature and difficult to apply. Currently, the blockchain applicationa to food safety is limited to traceability, and issues such as data integrity and data governance still need research.
- **Improved supply-chain decision-making.** There is still a need to help farmers make effective decisions in Agriculture 4.0, maintain effective connections among different complex networks, and identifythe dynamic needs of stakeholders.

### 2.3. Media Data

Social media has become the main way for users to obtain and share food information [27,28]. According to a statistical report on China’s Internet Network Information Center, the number of Internet users in China has reached 854 million, and 88.8% [29]. Social networks have gradually become the mainstream platform for disseminating information, a constant strem of videos, news, and other types of data [30]. Purcell et al. [31] found that two-thirds of Internet users get their news from Facebook and share news through social media. Through research into the generation, and promotion of social events on the Internet, the mode and characteristics of information transmission can be discovered, which provides support for practical application scenarios. In 2009, Google successfully predicted the spread of the H1N1 virus based on query data in its search engine and brought the public valuable time to prevent an outbreak [32]. Combining the real-time advantage of big data with the conventional and available advantages of traditional data will enable effective response to the transmission of public health events such as COVID-19 [33]. Singh et al. discovered supply chain management problems by using Twitter data to improve supply chain management in food industry [34].

The public participates in the discussion of events by different media, and it expresses clear opinions and attitudes in the form of public opinion [35]. The report (https://baijiahao.baidu.com/s?id=1617643364060321280&wfr=spider&for=pc (accessed on 2 September 2021)) shows that food safety and food rumors were first among hot food events in 2018, and the topic has become one of the prime targerts for media rumors, and social media’s intensification of rumors can create a widespread crisis [35]. By analyzing and understanding the trend of food incidents based on social media data, regulators can formulate timely countermeasures like enhancing public awareness through science education and shaping public opinion [36,37,38]. However, the field of media data has its challenges that need to be overcome.

- **Multi-source heterogeneous data fusion.** Media data on Twitter, Facebook, YouTube and various information portals have complex formats and multiple sources, and there is a lack of technology to identify relevant data in one sources and link it to others. In the absence of a "fusion technology" for multi-source heterogeneous data further study is urgently needed to address social media rumors and their negative impact on public security.
- **Rumor detection.** Network rumors seriously impair the public’s ability to recognize authentic of network information. The generation, influence, and propagation mechanism of network rumors have been studied [35,36,37,38], but there is still no answer to the problem of improving the public’s abiity to evaluate network information. The ability to perceive a risk has a positive impact on users’ attitudes, but social media undermines it. Authoritative information on refuting rumors, such as government notices and mainstream media news, have a significant effect on reducing the public’s acceptance of network rumors and improving the willingness to identify network rumors.
- **Rumor control.** Many studies based on communication science and psychology analyze food rumors by the way they are transmitted, but there is a lack of relevant research on government management. Existing food rumors detection technology is mostly simulation, which rarely considers the responsibilities of government institutions. It will be necessary to consider the role of the government, social media, and human networks to establish an efficient, and authoritative information platform to dispel rumors in time. This will improve public risk and prevention awareness, strengthen and punishment mechanisms for rumormongers, and strengthen legal education and behavioral guidance for the public [35]. The study of social network interaction patterns will be used to explore how to promote and change the public’s perception, attitude, and behavior on rumors concerning food, health or other fields.

## 3. Application of Big Data in Food Industry

**Figure 3:**
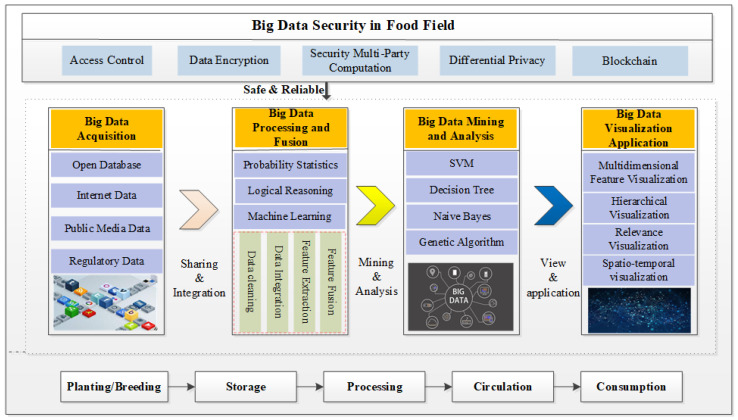
The processing model of big data in food.

The food supply chain is from farm to table, where the main links are planting and breeding, storage, processing, circulation (transportation) and consumption [39]. The discovery of value information on original data needs to go through a continuous cycle: of “discrete data—integrated data—knowledge understanding—mechanism extraction—application effect analysis”, from which the potential value of data sets can be mined. The processing system of big data application in food industry is shown in Figure 3. It is composed of five modules: big data collection of food industry, big data processing and fusion, big data mining and analysis, big data view and big data security. Each module is closely connected, and its functions are briefly described as follows.

- **Big data collection of the food industry.** Based on sensors, web crawlers, near-infrared detection instruments, food-related data is collected from different sources.
- **Multi-source data processing and fusion.** The collected data contaiins a considerable anount of redundant and dirty data [40], but through cleaning and conversion they can be removd and the data can be put into a standardized format. Then data features are extracted, and fusion is performed using probability statistics [41], logical reasoning and machine learning [42].
- **Big data mining and analysis.** This is a discovery mode of mining valuable knowledge from massive data that can accuratetly predict activities through scaled data [43]. Some big data technology, including SVM(Support Vector Machine) [44], Random Forest [45] and Naive Bayes [46], are used to analyze fused dataset to discover potential patterns to create social value [47,48].
- **Big data view.** Due to the characteristics of the complexity and multidimensional of data, it is necessary to generate a data view that can be easily expressed and understood by users. These are usually parallel coordinate, scatter graph, and scatter graph matrix methods [49,50].
- **Big data security.** In the lifecycle of big data on food, there are security risks in each processing stage [51], so research into big data security technology has become an important research topic. This module provides security technical support for all big data processing to ensure the safety, reliability, and controllability of data.

### 3.1. Social Co-Governance in the Food Industry

Social co-governance in the food industry provides a feasible solution to the issues of food security and food quality by using public wisdom [52]. Social co-governance is usually based on crowdsourcing to cooperate with consumers or experts to create value [53]. The failure rate of new food product development exceeds 40%, and the failure of new products usually affects the continued operation of small and medium-sized enterprises [3]. Large food companies have tried to collect consumer preference data through crowdsourcing, and have decided the direction of product development based on an analysis of big data. The Danone company encouraged consumers to vote for a creamy dessert flavor, and the 400,000 participants in 2006 more than doubled to 900,000 in 2011. Lay’s used the wisdom of crowds to develop more than 245,825 flavors of potato chip [54]. Procter & Gamble, Starbucks and Unilever sought better product design based on collective intelligence [55]. Employees often have a wealth of heterogeneous expertise, and companies can gain insight from their workforce to help improve economic performance. In addition, crowdfunding is another form of social co-governance, sharing business risks and alleviating capital pressure through mutual assistance [56]. Social co-governance has great potential for food security. Combining the mobile data of consumer groups with food shelf life, the intelligent control of food inventory can be realized to prevent food spoilage and waste [57]. Social co-governance can also be applied to monitoring foodborne diseases [32], identfying contaminated products, reducing the risk rate of food rumors and enhancing food safety [58].

Although social co-governance can enable food enterprises to obtain consumer demand information through diversified channels, it is still difficult to obtain effective information in time due to the limitation of enterprise resources [53]. In addition, there is a lack of an incentive or fair evaluation method in the food industry to convince consumers to participate.

### 3.2. Exploit Consumption Markets

There is a huge amount of food-related data both inside and outside the food supply chain, and the collection and analysis can promote enterprises to expand their markets [59]: (1) By collecting commodity and retail information for analysis, they can appraise the market situation, grasp the business dynamics of their competitors, and define the market positioning of products, thereby grasping market opportunity. (2) Collecting consumer information (purchase lists and channels, commodity preferences, usage cycle, family information, working condition, values) will establish a customer database that can give enterprises portraits of their customers that reveal their preferences, consumption tendency, value orientation and commodity reputation. With this information, enterprises can develop efficient marketing strategies and develop trust, so they can continue to compete effectively. (3) Data clustering analysis of consumers’ food evaluations (advantages and disadvantages, quality, nutritional value) from social platforms such as Facebook, Twitter and Sina Weibo, allows enterprises to anticipate potential problems and optimize the quality of goods and services.

### 3.3. Quantitative Production

By predicting future commercial demand based on historical sales’ data, agricultural and livestock products can be planned to reduce the probability of “cheap vegetables hurting farmers”. In addition, big data analysis can help predict weather more accurately, helping farmers and herdsmen to prepare for natural disasters. Analyses based on consumption and crop growth data help farmers decide which crops varieties to increase and which to reduce, improve crop yield, facilitate rapid sales, and achieve a return on capital. Big data can also optimize grazing area for local herdsmen and improve the usage rate of pasture. Fishermen can scientifically arrange fishing moratoria and locate fishing areas based on the results of big data analysis.

Consumption trends and habits provided by big data, enable governments to provide accurate guidance for agricultural and animal husbandry production, suggest production levels according to demand, and avoid unnecessary waste of resources and social wealth caused by excess capacity. When combined with drones, big data can promote the development of precision agriculture by allowing farmers to collect information on the growth of crops, diseases, and pests, at a much lower cost and with much higher accuracy than by hired aircraft [60].

### 3.4. New Dishes New Experience

In 1992, chefs Heston Blumenthal and Francois Benzi were deciding which ingredients with similar flavors would work well together, when someone created a combination of white chocolate and caviar. Due to the chemical differences, it tasted terrible. Today, because of food sciecne, there is a large amount of information about food chemicals [61] and how they make food taste. Consequently, Ahn et al. [62,63] developed a flavor network of ingredients connected by shared flavor compounds, in which flavors were limited by the type of raw materials. Garg et al. developed a flavor database with richer food materials [64]. Simas et al. promoted a flavor network and constructed the food-bridging network [65]. However, some well-known food combinations such as red wine and beef, do not share chemical compositions or flavor compounds, yet they are still very popular. Therefore, food pairings need to be seen in a broader spctrum, not just based on flavor compounds or chemical composition.

The future of food flavor design could be traced to 2019 when the company McCormick partnered with IBM to use Artificial Intelligence and big data to generate new flavor combinations by analyzing data from millions of datasets to meet the changing consumer demand.

### 3.5. Take-Out Service

In China, the number of online takeout users accounts for more than 44% of sales, and the scale has exceeded 398 million people (https://www.qianzhan.com/analyst/detail/220/200512-65621d53.html (accessed on 2 September 2021)). The take-out markets with its large number of users and rapid growth has generated a huge amount of takeout data. The takeout big data service platform not only helps the government supervise the industry, but also creates huge economic and social value. First, it predicts and informs customers of the delivery time, thereby avoiding disrupting consumers’ daily plans and helping restaurants establish a good reputation. Second, it helps the take-out enterprise understand consumer demand. Third, the take-out big data platform promotes the transparency of the supply chain, which is conducive to establishing and improving customers’ trust. Fourth, the overall running of the city can be clearly understood by analyzing the take-out dataset [66].

Since take-out data involves sensitive private information (the customer’s location, preference, bank, identity, and communication), ensuring data security in the take-oout big data platform is a serious challenge.

### 3.6. Precise Nutrition and Health Management

The development of big data provides technical support for the processing of massive data, and scientific guidance for human nutrition and health management. In the past, people usually learned nutrition information from experts, books, and the Internet. However, there was a lack of accurate nutrition and health management for individuals because of the difference in individual health conditions [67]. In an example of applying big data to the people’s daily diet Teng et al. proposed to use a recipe recommendation algorithm to determine which food ingredients were necessary [68]. Grace et al. combined case-based reasoning and a deep learning algorithm to generate new recipes [69,70]. However, it may also generate "dark cuisine" due to the the uncertain factor of deep learning. Some scholars, like Freyne et al., focused on a diet therapy. They developed a personalized recipe recommendation system for obese people based on the suggestions from medical professionals and research on obese people [71]. In anoher instance, Yoshida et al. proposed a personalized recipe recommendation based on users’ food preferences [72]. Zeevi et al. broke with traditional experience-based nutritional recommendations by using machine-learning algorithms to combine data (e.g., blood parameters, dietary habits, and gut microbiota) to formulate personalized diets that optimize postprandial glucose levels and metabolites [73]. The combination of big data with Artificial Intelligence will provide a new approach for the research of precision nutrition.

## 4. Challenges

While the food supply chain can benefit from big data, the following challenges need to be addressed.

- **Low data collection efficiency and poor data quality.** Due to the lack of effective data collection technology, there are problems with applying big data to food: missing or insufficient data, and difficulties with data forensics. Therefore, it is of great significance to study the collection and verification methods of multisourced big data on food. In the future, edge computing technology will be used to link the food supply chain thereby superseding traditional collection, which is hard to apply to a complex collection environment and collection requirements. Intelligent web crawler technology can be used to collect public opinion data from the Internet, and associate it with data from the physical world to form a complete big data view of food safety. It will solve the dilemma of the separation between physical world and public opinion data found in traditional methods.
- **Data islands.** There is one are in the food industry where data is not shared: government. Since regulatory data usually involve state secrets, and enterprise data may involve trade secrets, they reluctance to share these data seriously hinders the application and promotion of big data. Moreover, the independent management systems of enterprises and government creates poor system compatibility, which inhibits data circulation. Therefore, a new business model is urgently needed, for example: the establishment of an incentive mechanism to explore new methods of data fusion to establish an effective privacy protection method and encourage data owners to share data.
- **Data security.** Private information, such as consumer activity and social relationships, is hidden in food data. For example, based on the user’s location, there are connections to tracking data (such as order and logistical data and a location picture), so consumers can be tracked through association analysis, which may represent a risk to the their security [74]. In the life cycle of big data, there are security threats and privacy leakage risks at each processing stage [51,75,76]. The security protection analysis of big data for food is shown in Table 2. Data encryption is one of the effective methods for protecting data security. Rivest [77] proposed a privacy homomorphic encryption that would delegate part of a complex operations to a third party, directly operating on the ciphertext, who would return the results without disclosing of information. Yao et al. [78] proposed a secure multiparty computing scheme (SMC), in which all participants realize collaborative computing, and the privacy of all parties is protected, which eliminates the need for trusted third parties. However, ciphertexts have a serious impact on data readability. The strongly robust blockchain has anti-network attack, anti-eavesdropping, anti-tampering and anonymity features, that can protect the security and reliability of data [79]. Combining blockchain with an access control algorithm, Uchibeke et al. [80] proposed that data owners independently manage their data and prevent data leakage. However, blockchain technology still needs to be studied for security and work efficiency [81]; thus, research into big data security and privacy protection technology has become an important research area in the food field.

- **Security risks of crowdsourcing services.** On one hand, food enterprises have a large amount of enterprise data, but it is difficult to discover the potential value of the information. On the other hand, in crowdsourcing, consumers are not enthusiastic enough to participate, and there is a risk that the crowd will get out of control. In the take-out market, unlicensed crowdsourcing deliverers have become the biggest uncontrollable factor in takeout distribution, and it is a serious risk for take-out food safety. (1) Since the equipment of deliverers is self-regulated, it is difficult to control whether delivery safety standards can be achieved. (2) It is difficult to guarantee the hygiene and health of the people involved in the delivery. If a deliverer carries an infectious disease or the food is contaminated on the way, food safety cannot be guaranteed at all. (3) Take-out deliverers increase the management difficulty of the platform. Recently, the platform can only discipline deliverers through a user account ban. But the deliverer only needs to register with a new mobile phone number and borrow a person’s ID card for real name authentication, and then he can continue to deliver. Therefore, the establishment of a fair, incentive, and perfect crowdsourcing service mechanism will be one of the important research topics.
- **Healthy and sustainable development of the food industry.** By 2050, the world’s population will exceed 9 billion, and only through a healthy and sustainable food industry can global food demand be met. [89]. However, resource waste and foodborne diseases are key factors restricting this sustainable development: excessive chemical residue in crops from the of chemical fertilizers and pesticides [90]; perishable food loss in developing countries and enormous food waste in developed countries [91]; high energy consumption and pollution from food processing and transportation; and the discarding of potentially contaminated food because of the iinability to quickly and efficiently trace the source of a contamination. Therefore, the economic and environmental sustainability of stakeholders are the core factors for promoting the sustainable development of the food industry chain.

## 5. Conclusions

Because big data can provide a large amount of effective business information, the development of data-driven industries has attracted attention from all countries [92]. In 2012, the United States promoted big data as a national strategy to promote the formation of new economic growth and enhance national competitiveness [93]. Subsequently, EU member states formulated big data development strategies to transform traditional national governance models [94]. Big data to promote the development of the food industry has become a main research topic. This paper introduced the application of big data in food industry and showed that the main data sources are regulatory, enterprise, and media data. The results showed the great potential for big data for the food industry. Big data has particularly broad application prospects in social co-governance of the food industry, quantitative production, exploitation of consumption markets, new dishes, take-out services, and precise nutrition and health management. But, to exploit this full potential of big data, technical, social, and health and sustainable development issues require further research.

## Figures and Tables

**Table 1:** The public regulatory database.

Database	Database Type	Data Description	Country	Organization	Link/Source
Import and export food sampling	Alerts/notifications	Results of food sampling	China	NMPA	https://www.cfsn.cn/
Food sampling report	Alerts/notifications	Food sampling report	USA	FSIS	https://www.fsis.usda.gov/science-data/sampling-program
European Food Consumption Database	Alerts/notifications	European food consumption habits	European	EFSA	https://www.efsa.europa.eu/en/data-report/ efsa-food-composition-db
Import food sampling	Alerts/notifications	Results of food sampling	Japan	MHLW	https://www.mhlw.go.jp/content/11135200/000663987.pdf

**Table 2:** Analysis of data security protection technology in the whole life cycle of food data.

Life Cycle	Challenges	Protection Technology
Data collection	Data corruption, data loss, data leakage and data forgery	Data encryption [82]
Data storage	Illegal intrusion and data disclosure	Storage encryption [83], blockchain [79]
Data transmission	Data leakage and data corruption	Data encryption [84], privacy protection [85], blockchain [81]
Data usage	Information leakage and data abuse	Access control [86], SMC [78], data encryption [77], differential privacy protection [87]
Data Destruction	Privacy disclosure, destruct the data media	Data trusted deletion [88]

## Data Availability

Data sharing is not applicable for this article.
